# Ensemble Linear Subspace Analysis of High-Dimensional Data

**DOI:** 10.3390/e23030324

**Published:** 2021-03-09

**Authors:** S. Ejaz Ahmed, Saeid Amiri, Kjell Doksum

**Affiliations:** 1 Department of Mathematics and Statistics, Brock University, St. Catharines, ON L2S 3A1, Canada; sahmed5@brocku.ca; 2 Department of Civil, Geologic and Mining Engineering Polytechnique Montreál, Montreál, QC H3T 1J4, Canada; 3 Department of Statistics, University of Wisconsin, Madison, WI 53706, USA; doksum@cs.wisc.edu

**Keywords:** ensembling, high-dimensional data, Lasso, elastic net, penalty methods, prediction, random subspaces

## Abstract

Regression models provide prediction frameworks for multivariate mutual information analysis that uses information concepts when choosing covariates (also called features) that are important for analysis and prediction. We consider a high dimensional regression framework where the number of covariates (*p*) exceed the sample size (*n*). Recent work in high dimensional regression analysis has embraced an ensemble subspace approach that consists of selecting random subsets of covariates with fewer than *p* covariates, doing statistical analysis on each subset, and then merging the results from the subsets. We examine conditions under which penalty methods such as Lasso perform better when used in the ensemble approach by computing mean squared prediction errors for simulations and a real data example. Linear models with both random and fixed designs are considered. We examine two versions of penalty methods: one where the tuning parameter is selected by cross-validation; and one where the final predictor is a trimmed average of individual predictors corresponding to the members of a set of fixed tuning parameters. We find that the ensemble approach improves on penalty methods for several important real data and model scenarios. The improvement occurs when covariates are strongly associated with the response, when the complexity of the model is high. In such cases, the trimmed average version of ensemble Lasso is often the best predictor.

## 1. Introduction

Recent research in statistical science has focused on developing effective and useful techniques for analyzing high-dimensional data where the number of variables substantially exceeds the number of cases or subjects. Examples of such data sets are genome or gene expression arrays, and other biomarkers based on RNA and proteins. The challenge is to find associations between such markers (*X*’s) and phenotype (*Y*).

Regression models provide useful frameworks for multivariate mutual information analysis that uses information concepts when choosing covariates (also called features) that are important for the analysis and prediction. A recent article that includes both the concept of mutual information and the Lasso is [1]. This paper develops properties of methods that use the information in a vector *X* to reduce prediction error, that is, to reduce entropy. We consider regression experiments, that is, experiments with a response variable Y∈R and a covariate vector ${\left(X_{1},\ldots ,X_{p}\right)}^{t}$. The objective is to use a sample of i.i.d. vectors $(x_{i},y_{i}),1\leq i\leq n$, where $x_{i}={\left(x_{i1},\ldots ,x_{ip}\right)}^{t}$ with $x_{ij}\in R$, to construct a predictor ${\hat{Y}}_{0}$ of a response $Y_{0}$ corresponding to a covariate vector $x_{0}={\left(x_{01},\ldots ,x_{0p}\right)}^{t}$ that is not part of the sample. Let $X={\left(x_{ij}\right)}_{n\times p}$ be the design matrix of explanatory variables (covariates) and $y={\left(y_{1},\ldots ,y_{n}\right)}^{t}$ be the vector of response variables. Denote X[,j] as the *j*th column vector of the design matrix. We will use the linear model

(1) y=Xβ+ϵ,

where $\beta ={\left(\beta_{1},\ldots ,\beta_{p}\right)}^{t}$ is the vector of regression coefficients and $\epsilon ={\left(\epsilon_{1},\ldots ,\epsilon_{n}\right)}^{t}$∼$N(0,\sigma^{2}I)$ is the residual error term. In this model, predictors ${\hat{Y}}_{0}$ take the form

$${\hat{Y}}_{0}=\sum_{j=1}^{p}{\hat{\beta }}_{j}x_{0,j},$$

where ${\hat{\beta }}_{j}$ is an estimator based on the i.i.d. sample $(x_{i},y_{i}),1\leq i\leq n$.

Under n≥p, the ordinary least square (OLS) estimator of β can be used. When n<p a unique OLS estimate does not exist. However, for sparse models where most of the β’s are zero, we can use the Lasso [2] criteria that forces many of the estimated β’s to be set to zero. For a given penalty level λ≥0, the Lasso estimate of β is

$$\begin{array}{c} \hat{\beta }={\mathrm{argmin}}_{\beta }\left\{\frac{1}{2}{∥y-x\beta ∥}_{2}^{2}+\lambda {∥\beta ∥}_{1}\right\}, \end{array}$$

where ${∥.∥}_{2}$ is the Euclidean distance and ${∥\beta ∥}_{1}=\sum \left|\beta_{j}\right|$ is the $\ell_{1}$-norm. The Lasso not only sets a subset of β’s to zero, it also shrinks OLS estimates of the remaining β’s towards zero. It is an effective procedure for experiments when one can assume that the number *r* of covariates that are relevant for the response in the sense that their β coefficient is not zero, satisfies r≤n. That is, for sparse models.

Other effective high-dimension methods that we consider are adaptive Lasso, ref. [3], smoothly clipped absolute deviation (SCAD), ref. [4], least angle regression (LARS), ref. [5], and elastic net, ref. [6]. The properties of Lasso, and its variants, are well studied to examine consistency of parameter estimates [7,8], and to assess the prediction error and the variable selection process [9,10] examined properties of the Lasso in partially linear models. Several variants of Lasso were introduced by [11] and more recently by [12]. See [13,14,15] for many of the extensions of the original Lasso.

In this paper, we examine properties of statistical methods based on Ensemble Linear Subspace Analysis (ELSA) for analyzing high-dimensional data. ELSA is based on repeated random selection of subsets of covariates, doing statistical inference on each of the subsets, and then combing the results from subsets to construct a final inference. One advantages of this ensemble subspace approach is that it makes the analysis of studies with a million or more covariates variables more manageable. Another advantage is that for many situations the ensemble approach is more efficient because it takes advantage of the high efficiency of statistical methods for the case where the number of covarites is less than or equal to the sample size.

Classical examples using sub-models whose results are pooled and aggregated into a final statistical analysis is the bagging method ([16]) and the random forests approach ([17]). Recent studies that use ensemble ideas include [18,19]. These papers focus on feature selection, that is, selecting the covariates that are associated with the response variable. This paper deals with using the selected covariates to construct efficient predictors of the response. We examine conditions under which penalty methods such as Lasso perform better when used in the ensemble approach by computing mean squared prediction errors for simulations and a real data example. Linear models with both random and fixed designs are considered. We examine two versions of penalty methods: one where the tuning parameter is selected by cross-validation; and one where the final predictor is a trimmed average of individual predictors corresponding to the members of a set of fixed tuning parameters. We find that the ensemble approach improves on penalty methods for several important real data and model scenarios. The improvement occurs when covariates are strongly associated with the response, when the complexity of the model (represented by r/p) is high. In such cases, the trimmed average version of ensemble Lasso is often the best predictor.

The rest of this article is organized as follows. In Section 2 and Section 3, we introduce six different approaches to subspace selection. Section 3 describes a new approach for dealing with tuning parameters λ. Instead of using the standard Lasso based on a $\hat{\lambda }$ obtained by cross validation, it computes Lasso predictors for a fixed set of tuning parameters and uses the average of these predictors as the fixed predictors. Section 4 outlines other penalty-based ensemble methods for high dimensional data. Section 5 introduces the concepts of mean squared Prediction Error (MSPE) and efficiency (EFF) for fixed and random design experiments as well as for real data. Section 6 gives efficiency of various penalty methods with respect to CV Lasso, including efficiencies of ensemble subspace version of these penalty methods. The efficiency results show that when the model complexity r/p is moderately high, trimmed subspace method perform best in all but one case. Section 7 compares six ensemble subspace Lasso methods to the standard CV Lasso. For models with a mixture of strong and weak signals, the ensemble methods perform best except when the models are very sparse. The final section gives a summary of results.

## 2. Ensembling via Random Subspaces

The following three-step protocol provides the ensemble subspace approach:

- Divide the initial dataset (X,y), $X={\left(x_{ij}\right)}_{n\times p},y\in R^{n}$ randomly into smaller subdatasets by selecting at random subsets covariates. The sample size *n* remains the same.
- Construct predictors of the future response $Y_{0}$ within each sub dataset.
- Combine the results obtained from each sub dataset into a final analysis.

We consider three approaches to choosing subsets of X-variables

1. Choose subspaces with $p^{*}$ covariates, where $p^{*}$ is the number of distinct covariates after randomly selecting *p* covariates with replacement from the collection of all covariates. Here the random variable $p^{*}$ is known to have expected value approximately 0.63*p*. Let $x^{*}$ denote the distinct covariates and $X^{*}$ denote the corresponding design matrix. The subspace data is $\left(X^{*},y\right)$ where $y\in R^{n}$ and $X^{*}={\left(x_{ij}^{*}\right)}_{n\times p^{*}}$. By repeating this procedure *B* times independently and using a method such as Lasso we get predictors $\left\{{\hat{Y}}_{0,1},\ldots ,{\hat{Y}}_{0,B}\right\}$.

2. Choose *n* covariates without replacement from the *p* covariates, repeating *B* times independently and using a method such as Lasso thereby obtaining $\left\{{\hat{Y}}_{0,1},\ldots ,{\hat{Y}}_{0,B}\right\}$.

3. Same as 2., except choose n/2 covariates.

The final prediction of the response based on a covariate vector $x_{0}$ is ${\hat{Y}}_{0}\left(x_{0}\right)=B^{-1}\sum_{b=1}^{B}{\hat{Y}}_{0b}\left(x_{0}\right)$. Note that the terms in the sum that defines ${\hat{Y}}_{0b}\left(x_{0}\right)$ are identically distributed, but not independent. Thus, with ${\hat{Y}}_{0}={\hat{Y}}_{0}\left(x_{0}\right)$ and ${\hat{Y}}_{0b}={\hat{Y}}_{0b}\left(x_{0}\right)$

(2) $$\begin{array}{c} Var\left({\hat{Y}}_{0b}\right)=\frac{1}{B}Var\left({\hat{Y}}_{01}\right)+\frac{B-1}{B}Cov\left({\hat{Y}}_{01},{\hat{Y}}_{02}\right)=\rho \sigma^{2}+\frac{1-\rho }{B}\sigma^{2}, \end{array}$$

where $\sigma^{2}$ is the variance of one predictor ${\hat{Y}}_{0}$ and ρ is the pairwise correlation between two such predictors. By selecting *B* large, we can make the second term negligible. When ρ is sufficiently small $\rho \sigma^{2}$ can in many cases be smaller than the variance of the predictor based on all the covariates. When ${\hat{Y}}_{0}$ is prediction unbiased, that is, $E({\hat{Y}}_{0}-Y)=0$, then $Var({\hat{Y}}_{0})$ equals the prediction mean squared error (PMSE). When the subspace have *n* or fewer variables, OLS is prediction unbiased.

## 3. Prediction on Subspaces

We consider two approaches for dealing with Lasso tuning parameters: the cross-validated and the Trimmed Lasso. The same approaches will be applied to the other penalty methods. Let $X^{*}=\left\{x_{ij}^{*}\right\}$ be the subspace design matrix. The Lasso estimate based on a linear model on the subspace is

$$\begin{array}{c} \hat{\beta }={\mathrm{argmin}}_{\beta }\left\{\frac{1}{2}∥y-X^{*}{\beta ∥}_{2}^{2}+\lambda {∥\beta ∥}_{1}\right\}, \end{array}$$

The standard procedure is to choose the tuning parameter λ using 10-fold cross-validation (CV), which denoted as CVLasso hereafter. Note, since the size of subspace design $X^{*}=\left\{x_{ij}^{*}\right\}$ is changed, $\hat{\beta }$ is changed as well and correspond the number variables in $X^{*}=\left\{x_{ij}^{*}\right\}$. It is implemented in the library “glmnet” in R. Cross validation may sometimes lead to unfortunate choices of λ because the random choices of training and test sample may not yield a λ that represents a λ that will give a good predictor. Thus we will consider a method based on a collection of fixed λ’s. This method, which we call the *Trimmed Lasso (TrLasso)*, uses as predictor the trimmed average (10% in each tails) of Lasso predictors computed from a path of 100 λ’s. The path is generated using the library glmnet in R with option “nlambda”. The largest lambda, $\lambda_{MAX}$, is the smallest value for which all beta coefficients are zero while $\lambda_{MIN}=\lambda_{MAX}e^{-6}$. The λ values are equally spaced on the log scale. We consider six versions of ensemble subspace methods. In the following, “approach *j*” for j=1,2 and 3 chooses subspace sizes $p^{*}$, *n*, and n/2, respectively.

ETrLasso (j): For j=1,2 and 3 use approach (j) to choose the number of variables in each subspace. Then apply TrLasso in each subspace.
ECVTLasso (j): For j=1,2 and 3 use approach (j) to choose the number of variables in each subspace. Then apply CVLasso in each subspace.

## 4. Competitors to Lasso

### 4.1. Elastic-Net

For highly correlated predictor variables the Lasso tends to select a few of them and shrink the rest to zero, see [6,15] for an extensive discussion. For such cases the Elastic Net, denoted ELNET hereafter, is suggested as a compromise between the ridge and the Lasso methods. The estimates of coefficients can be obtained from:

(3) $$\begin{array}{c} \hat{\beta }={\mathrm{argmin}}_{\beta }\left\{\frac{1}{2}{∥Y-X\beta ∥}_{2}^{2}+\lambda \left(\frac{1}{2}{\left(1-\alpha \right)∥\beta ∥}_{2}^{2}+\alpha {∥\beta ∥}_{1}\right)\right\}, \end{array}$$

where α∈[0,1]. Here α=1 leads to the regular Lasso. The penalty parameters, λ and α, are two nonnegative tuning parameters.

We examine properties of ELNET using of α = 0.25, 0.5, and 0.75, while λ is treated as for the Lasso. Thus we obtain TrELNET(α) and CVELNET(α). For ELNET the ensemble subspace method is also carried out as for the Lasso but only using the trimmed (10%) option, resulting in three methods for each α. We use the notation TrELNET(j,α) and ELNET(j,α), j=1,2,3 for the trimmed and CV ensemble subspace option for subspace of size $p^{*},n,$ and n/2. The calculations of these ELNETs, including the Lasso where α=1, are done using the library glmnet in R.

### 4.2. Adaptive Lasso

Ref. [3] introduced the adaptive Lasso for linear regression. It uses a weighted penalty of the form $\sum_{j=1}^{p}w_{j}\left|{\hat{\beta }}_{j}\right|$ where $w_{j}=1/\left|{\hat{\beta }}_{j}\right|$ and ${\hat{\beta }}_{j}$ is a preliminary estimate of $\beta_{j}$ and

(4) $$\begin{array}{c} \hat{\beta }={\mathrm{argmin}}_{\beta }\left\{\frac{1}{2}{∥Y-X\beta ∥}_{2}^{2}+\lambda {∥w\beta ∥}_{1}\right)\}. \end{array}$$

The preliminary beta estimate is typically the Ridge estimate. We use that in our simulation studies. The Adaptive Lasso is also computed as a 10% trimmed average of Lasso predictors for a sequenced of λ’s and as the predictor obtained when λ is selected using CV. They are denoted as TrALasso and CVALasso, respectively. We consider these methods for the proposed ensembled subspace procedures and denote them as ETrAlasso(*j*) and ECVAlasso(*j*), j=1,2,3.

### 4.3. Lars

Least angle regression, also called LARS, was developed in [5]. It uses a model selection algorithms based on forward selection that enables the procedure to select a parsimonious set of predictors to be used for the efficient prediction of a response variable from an available large collection of possible covariates. It improves computational efficiency compared to the Lasso. As in Section 3, LARS is considered with trimming and with CV in prediction. They are denoted as TrLARS and CVLARS, respectively. We consider the trimmed and CV versions of these methods for the proposed ensembled subspace procedure and denoted them as ETrLARS(*j*) and ECVLARS(*j*), j=1,2,3. The calculation of LARS is done by using the library LAR in R.

### 4.4. Scad

Ref. [4] introduced the SCAD penalty for linear regression. It is a symmetric and quadratic spline on the reals whose first order derivative is

(5) $$\begin{array}{c} SCAD_{\lambda ,a}^{\prime }\left(x\right)=\lambda \left\{I\left(|x|\leq \lambda \right)+\frac{(a\lambda -|x|)+}{(a-1)\lambda }I\left(|x|>\lambda \right)\right\}, \end{array}$$

where λ>0 and a=3.7 as recommended by [4]. The SCAD penalty is continuously differentiable and can produce sparse solutions and nearly unbiased estimates for sparce models with large beta coefficients. The CV and trimmed version of SCAD will be labeled as CVSCAD and TrSCAD, while the ensemble subspace methods will be ECVSCAD(*j*) and ETrSCAD(*j*), j=1,2,3.

## 5. Mean Squared Prediction Error (MSPE)

### 5.1. (a) Random Covariates, Simulated Data

To examine prediction error, we generate a training set $D=\{\left(x_{1},y_{1}\right),\ldots ,\left(x_{n},y_{n}\right)\}$ using the simulation model under consideration, and for each method considered obtain a predictor of the form ${\hat{y}}_{i}=\sum_{j=1}^{p}{\hat{\beta }}_{j}x_{ij},i=1,\ldots ,n$. To explore the performance of proposed methods on data not used in producing the prediction formula, we independently generate a test set $D_{0}=\left\{\left(x_{01},y_{01}\right),\ldots ,\left(x_{0n_{0}},y_{0n_{0}}\right)\right\}$ and compute

$$\mathrm{MSPE}=\frac{1}{n_{0}}\sum_{i=1}^{n_{0}}{\left(y_{0i}-{\hat{y}}_{0i}\right)}^{2},$$

where

$${\hat{y}}_{0i}=\sum_{j=1}^{p}{\hat{\beta }}_{j}x_{0ij},\;i=1,\ldots ,n_{0},$$

is the predicted value of $y_{0i}$ based on $x_{0i}$. We use $n_{0}=0.3n$ in the simulation studies. We repeat the process of generating independent collections for training and test sets M=2000 times, therby obtaining MSPE${}_{1},\ldots ,$ MSPE${}_{M}$. We measure the efficiency of a predictor $\hat{Y}$ by comparing it to the standard method, Lasso with cross-validation

(6) $$\begin{array}{c} EFF\left(\hat{Y}\right)=\frac{1}{M}\sum_{b}\frac{MSPE_{b}\left(\mathrm{CVLasso}\right)}{MSPE_{b}\left(\hat{Y}\right)}, \end{array}$$

where the sum is over the simulation, and as mentioned earlier for the Lass the standard procedure is to choose the tuning parameter λ using 10-fold cross-validation (CV).

### 5.2. (b) Fixed Covariate, Simulated and Real Data

Let $D=\{\left(x_{1},y_{1}\right),\ldots ,\left(x_{n},y_{n}\right)\}$, $x\in R^{p}$ and y∈R, denote a real or simulated data set with random *y*’s and fixed x’s. Split this set into a test set $D_{0}$ with $n_{0}$ data vectors and a training set $D_{1}$ with the remaining $n_{1}$ data vectors, where $n_{0}=0.3n$ and $n_{1}=0.7n$. For each of the discussed methods, the training set is used to produce a prediction algorithm that is used to predict the *y*’s in the test set. The MSPE is then $\mathrm{MSPE}=\frac{1}{n_{0}}\sum_{i=1}^{n_{0}}{\left({\hat{y}}_{0i}-y_{0i}\right)}^{2},$ where ${\hat{y}}_{0i}$ is the predicted value of $y_{0i}$ based on x’s in the test set. Next we compute the ratio with respect to CVLasso(MSPE). This procedure is repeated 2000 times and the average is the final EFF($\hat{Y}$). For simulated experiments, an additional M=2000 repetitions is carried out.

## 6. Efficiency Result for Lasso Competitors

In the following, we compare the accuracy of the methods presented in Section 3 and Section 4. The results are presented with B=250 subspaces; we also tried B=500, but since the result were nearly the same, they are not presented here. We examine the relative performance of the methods as a function of the complexity index which is defined as the ratio r/p of the number of covariates that are relevant for the response *y* to the total number of covariates.

### 6.1. Syndrome Gene Data

Ref. [20] studied expression quantitative trait locus mapping in the laboratory rat to gain a broad perspective of gene regulation in the mammalian eye and to identify genetic variation relevant to human eye disease. The dataset which is from the flare library in R has n=120 with p=200 predictors, it includes the expression level of TRIM32 gene which can be considered as dependent variable. To compare the accuracy of the proposed methods on this dataset, we randomly select 30% of the data as a test set and consider the rest as a training set, and calculate the relative efficiency EFF($\hat{Y}$) to CVLasso. We repeat the procedure of selecting training and test set 2000 times which provide good accuracy. The results are reported in Table 1.

Among the seven Lasso Type competitor to CVLasso, the most efficient in terms of EFF($\hat{Y}$) is the one based on subspaces of sizes n/2=60 and based on a trimmed average of Lasso predictors computed for a sequence of λ tuning parameters. We found that it improves on CVLasso 83% of the time. However, the average of the mean square prediction error ratios is EFF$(\hat{Y})=1.11$, thus the improvement does not appear to be substantial.

Turning to the other procedures in Table 1, we see that, generally, the best performance is obtained for the trimmed ensemble versions based on subspaces of size n/2, expect for adaptive Lasso which is best for subspace size *n*. Generally, the improvement ensemble over CvLasso is about 1.1 in terms of EFF($\hat{Y}$). Moreover, the performance of these methods are very close, including ELNET methods with different α. That is, using subspaces and a robust trimmed average of response predictors obtained from the path of glment lambdas is more efficient than using the predictor based on the lambda selected by glment cross validation. The improvement achieved by the trimmed ensemble versions of SCAD based on subspaces of size n/2 over the basic (CV and trimmed) versions of SCAD is striking.

### 6.2. Simulation Efficiency Results

We next used a modification of a model set forth by [21]. We set p=1000, and in contrast to the syndrome Gene inspired model, we now use i.i.d. random x’s, as indicated in Model (7). The model provides a large range of β values corresponding to strong, moderate and weak covariate signals. The correlations between covariates renage from 0.28 and 0.94.

(7) $$\begin{array}{ccc} & & X\overset{}{∼}N\left(M,\Sigma \right),\\ & & M={\left(\mu_{i}\right)}_{i=1,\ldots ,p},\;\;\mu_{i}\overset{i.i.d}{∼}N\left(5,2\right),\\ & & \Sigma ={\left(\sigma_{i,j}\right)}_{i,j=1,\ldots ,p},\;\;\sigma_{i,j}=\sigma_{j,i}\overset{i.i.d}{∼}Unif\left(0.4,0.6\right),i\neq j\\ & & \sigma_{i,i}∼Unif\left(0.8,1.2\right),\\ & & \beta_{j_{0}+1},\ldots ,\beta_{j_{0}+r}\overset{i.i.d}{∼}Unif\left(-2,2\right),\;j_{0}\in \left\{1,\ldots ,p-r\right\},\\ & & \beta_{j}=0,\;\mathrm{for}\;\mathrm{all}\;\mathrm{other}j,\\ & & y_{i}=\sum_{j=1}^{p}\beta_{j}x_{ij}+\epsilon_{i},\;\;\mathrm{with}\;\;\epsilon_{i}\overset{i.i.d}{∼}N\left(0,0.15\right),i=1,\ldots ,n. \end{array}$$

Using this model, we generate $\left(x_{1},y_{1}\right),\ldots ,\left(x_{n},y_{n}\right)$, n=180. Table 2, Table 3, Table 4 and Table 5 give the mean of the efficiency criteria over M=2000trials. The numbers in parentheses are standard deviations (SD). We next discuss the result for the case with r=150 relevant variables. Here *k* denotes the number of covariates in the subspaces, and $p^{*}$ is the number of distinct variables in a bootstrap sample from the set of covariates.

#### 6.2.1. Results for r/p=0.15

##### (a) Lasso Based Methods

Trimmed Lasso based on all p=1000 covariates performs best, with ensemble trimmed Lasso with $k=p^{*}$, a close second. Ensemble CVLasso performs poorly for all *k*. The trimming approach dominates the cross validation approach.

##### (b) ELNET Based Methods

CV and trimmed ELNET based on all p=1000 covariates are close and better than the ensemble methods and CVLasso. The value α in ELNET does not make much difference. Among ensemble methods, the trimmed version with $k=p^{*}$ and α=0.75 is the best, it is slightly better than CVLasso.

##### (c) LARS Based Methods

The trimmed and CV ensemble subspace methods with $k=p^{*}$ are best with the trimmed version slightly better. Both are better than CV Lasso.

##### (d) Adaptive Lasso Based Methods

CV ensemble adaptive Lasso based on subspaces with $k=p^{*}$ is best among all methods.

##### (e) SCAD Based Methods

For this model, SCAD does poorly for all but one version, presumably because it produces poor predictors for β’s that are close to zero. The one version that does well is the trimmed ensemble method with $k=p^{*}$ variables.

#### 6.2.2. Results for r/p=0.30

##### (a) Lasso Based Methods

Trimmed ensemble Lasso based on $p^{*}$ covariates in the subspaces performs best. The trimming approach outperforms the CV approach for each of *k*.

##### (b) ELNET Based Methods

Trimmed ensemble ELNET based on $p^{*}$ covariates performs best. The trimming approach outperforms the CV approach for each *k*. The value of α does not make much difference.

##### (c) LARS Based Methods

Trimmed ensemble LARS based on $p^{*}$ covariates is best among all LARS methods. Trimmed methods outperform CV methods.

##### (d) Adaptive Lasso Based Methods

CV Adaptive ensemble Lasso based on subspaces with $p^{*}$ covariates is best among all methods. Trimmed methods outperform CV methods except when $k=p^{*}$.

##### (e) SCAD Based Methods

Trimmed ensemble SCAD with $p^{*}$ covariates in the supspaces does well. Trimmed ensemble versions outperform CV version and the k=1000 version.

#### 6.2.3. Overall Summary

Table 2, Table 3, Table 4 and Table 5 show that the ensemble and trimming methods can improve on the CV Lasso. Overall, the CV esnsemble Adaptive Lasso based on subspaces with $p^{*}$ covariates performs best. For r/p=0.30, that is, 30% complexity, ensemble subsace with $p^{*}$ covariates does best overall and the trimmed approach is best except for the Adaptive Lasso. When r/p=0.15, the results are less clear, except the ensemble subspaces with $p^{*}$ covariates yields the overall best result when coupled with the Adaptive Lasso. The overall superior performance of ensemble subspace methods based on $p^{*}$ can in part be explained by formula (2) because the $p^{*}$ methods produce predictors that are weakly correlated.

## 7. Comparison of Cv and Trimmed Lasso Methods

### 7.1. Syndrome Gene Data Inspired Simulation Model

Simulation based on real data is very important from an application perspective, because the structure of the underlying population is often unknown. In this subsection, we use x from [20] as described in Section 6.1. That is we use non-random covariates to compare the efficiencies of the proposed Lasso-based methods on this dataset as a function of the complexity index r/p. We randomly selected *r* predictor variables from p= 200 predictors, where r/p ranges from 0 to and 0.5, and used the following models with *r* covariates relevant to the response *Y*.

(8) $$\begin{array}{ccc} & & \beta_{j_{0}+1},\ldots ,\beta_{j_{0}+r}\overset{i.i.d}{∼}Unif\left(-2,2\right),\;j_{0}\in \left\{1,\ldots ,200-r\right\},\\ & & \beta_{j}=0,\;\mathrm{for}\;\mathrm{all}\;\mathrm{other}\;j,\\ & & y_{i}=\sum_{j=1}^{p}\beta_{j}x_{ij}+\epsilon_{i},\;\;\mathrm{with}\;\;\epsilon \overset{i.i.d}{∼}N\left(0,0.4\right). \end{array}$$

**Figure 1:**
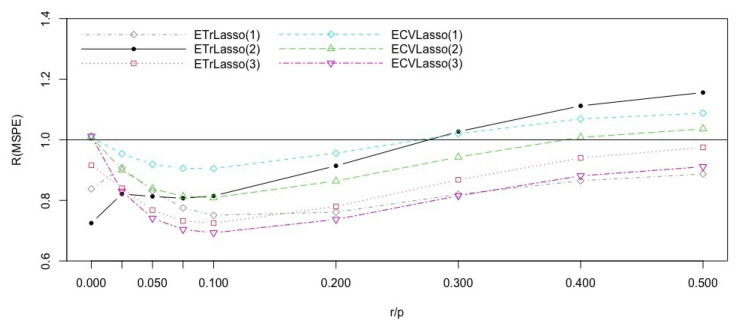
Efficiencies of the Lasso ensemble subspace methods with respect to the CVLasso for the Syndrome Gene inspired simulation model, with different complexity indices r/p.

The average of the standard deviations of the predictors is 0.28, so we considered ϵ∼N(0,0.4). We then calculated the discussed efficiencies of the proposed methods using M=2000. The result are reported in Figure 1. It shows that for r/p less than 0.29 the Lasso cross validated method has the best performance. For r/p larger than 0.29, the trimmed subspace version with *n* variables in the subspaces is best with cross validatioed ensemble Lasso with $p^{*}$ covariates a close second. This CV ensemble Lasso is also second best for r/p<0.29. For r/p<0.29, the performance of subspace methods are poor.

To summarize, in terms of predictor error, for sparse models, the cross validated lasso based on all covariates performs best, while for the model with r/p larger than 0.29, the trimmed ensemble lasso based on subspaces of size *n* performs best.

### 7.2. Simulated Models with Random Covariates

#### 7.2.1. (a) Strong and Weak Signals. Strong Covariate Correlations

**Figure 2:**
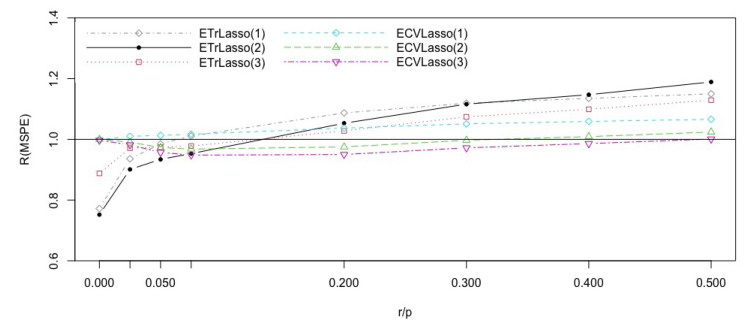
Efficiencies of the Lasso ensemble subspace methods with respect to the CVLasso for the model (7), with different complexity indices r/p.

We consider model (7) with values of r/p ranging from 0 to 0.5. The results in Figure 2 show that the ensemble CV Lasso based on subspaces with $p^{*}$ covariates improves on the CV Lasso for all values of the complexity index r/p. The ensemble trimmed Lasso with $p^{*}$ covariates is for best 0.07<r/p<0.3 while the ensemble trimmed Lasso with *n* covariates in each subspace is best for r/p>0.3. The ensemble CV Lasso’s with *n* and n/2 covariates are slightly worse than CV Lasso.

To summarize, the ensemble methods with $p^{*}$ covariates in the subspaces perform very well when compared to the CV Lasso. The ensemble trimmed Lasso versions are best for values of r/p larger than 0.2. This shows that when there are many covariates with strong and weak signals cross validation may lead to a poor choice of the trimming parameter λ.

#### 7.2.2. (b) Strong and Weak Signals. Weak Covariate Correlations

We consider model (7) with $\sigma_{ij}$ replaced by

(9) $$\begin{array}{c} \sigma_{ij}∼Unif\left(0.0,0.2\right). \end{array}$$

**Figure 3:**
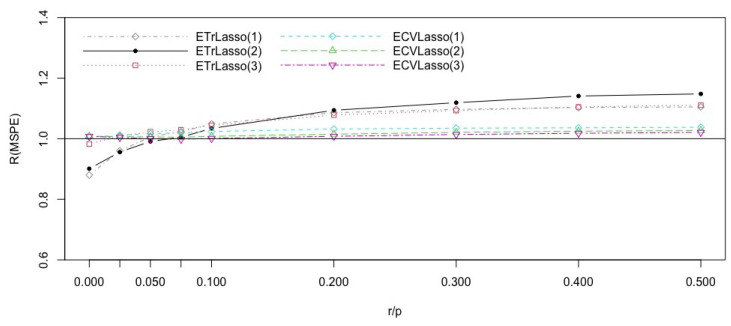
Efficiencies of the Lasso ensemble subspace methods with respect to the CVLasso for the model (9), with different complexity indices r/p.

Figure 3 shows that the dominance of the ensemble trimmed Lasso methods holds for r/p>0.09. In other words, when there is weak correlations between the covariates, and the complexity of the model is more than 0.09, it is better to use the trimmed average of ensemble predictors based ona sequence of fixed trimming parameters than using trimming parameters obtained by cross validation.

#### 7.2.3. (c) Strong Signals. Weak Covariate Correlations

We consider model (9) with β replaced by

(10) $$\begin{array}{c} \beta ∼Unif(2,3). \end{array}$$

**Figure 4:**
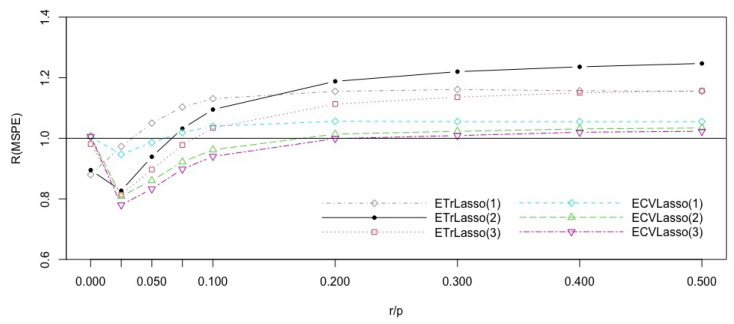
Efficiencies of the Lasso ensemble subspace methods with respect to the CVLasso for the model (10), with different complexity indices r/p.

Figure 4 shows that for very small complexity (r/p≤0.020), CV Lasso is best, while for r/p>0.020, the ensemble trimmed Lasso with $p^{*}$ covariates in the subspaces improves an CV Lasso and does very well overall. For r/p>0.15, the ensemble trimmed Lasso with *n* covariates in the subspaces is best. The trimmed ensemble versions do better than the CV ensemble versions for r/p>0.025.

#### 7.2.4. (d) Weak Signal. Weak and Strong Correlation between Covariates

These two cases had very similar results. Here we give only the case where we use model (9) with

(11) $$\begin{array}{c} \beta ∼Unif(-0.2,0.2). \end{array}$$

**Figure 5:**
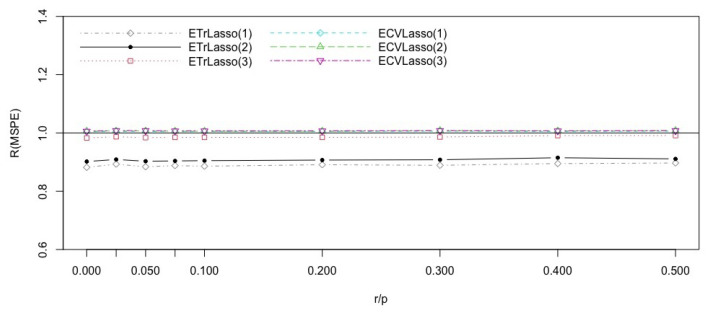
Efficiencies of the Lasso ensemble subspace methods with respect to the CVLasso for the model (11), with different complexity indices r/p.

Figure 5 shows that in this case the ensemble trimmed Lasso methods with $p^{*}$ and with *n* covariates in the subspaces do poorly. The ensemble CV Lasso methods performs at the same level as CV Lasso, as does the ensemble trimmed mean approach with k=n/2.

## 8. Conclusions

This article explores the random ensemble subspace approach for high-dimensional data analysis. This technique splits the data into covariate subspaces and generates models and methods on each covariate subspace. Merging and assembling the methods provides a global solution to the high-dimensional data analysis challenge. Let *n* denote the sample size and *p* the member of covariates, under p>>n. We consider three different approaches of selecting subspaces: repeatedly select subspaces as follows (1) *n* covariates with replacement from *p* covariates, then use the distinct covariates to form subspaces, (2) *n* covariates at random without replacement, and (3) n/2 covariates on random without replacement. This approach is applied to a variety of penalty methods and compared to cross-validation (CV) Lasso using mean squared predictor error (MSPE). We consider MSPE as a function of model complexity, which is defined as r/p where *r* is the number of covariates that are associated with the response and find that when r/p is moderate to large, the cross-validation ensemble subspace approach improves the CVLasso that uses all *p* covariates in one step. We also introduced an alternative to cross-validation that consists of computing predictors for a fixed set of data-based tuning parameters and using these predictors’ trimmed mean. This approach works well when the ratio r/p is above 0.2.

To facilitate communication among researchers and provide possible collaborations between scientists across disciplines and as supporters of open-science, the codes are written in R according to the end-to-end protocol we implemented in this manuscript, which are available on request.

## Figures and Tables

**Table 1:** Efficiencies with respect to CVLasso for the Syndrome Gene data.

Method				
CVLasso	TrLasso	ETrLasso(1)	ETrLasso(2)	ETrLasso(3)
-	1.048(0.002)	1.059(0.002)	1.079(0.002)	1.102(0.002)
		ECVLasso(1)	ECVLasso(2)	ECVLasso(3)
		1.056(0.001)	1.067(0.002)	1.059(0.002)
CVELNET(0.25)	TrELNET(0.25)	ETrELNET(1,0.25)	ETrELNET(2,0.25)	ETrELNET(3,0.25)
1.028(0.001)	1.057(0.002)	1.056(0.002)	1.092(0.002)	1.103(0.002)
		ECVELNET(1,0.25)	ECVELNET(2,0.25)	ECVELNET(3,0.25)
	1.068(0.001)	1.071(0.002)	1.059(0.002)	
CVELNET(0.50)	TrELNET(0.50)	ETrELNET(1,0.50)	ETrELNET(2,0.50)	ETrELNET(3,0.50)
1.014(0.000)	1.053(0.002)	1.059(0.002)	1.084(0.002)	1.103(0.002)
		ECVELNET(1,0.50)	ECVELNET(2,0.50)	ECVELNET(3,0.50)
		1.062(0.001)	1.069(0.002)	1.060(0.002)
CVELNET(0.75)	TrELNET(0.75)	ETrELNET(1,0.75)	ETrELNET(2,0.75)	ETrELNET(3,0.75)
1.006(0.000)	1.049(0.002)	1.059(0.002)	1.081(0.002)	1.103(0.002)
		ECVELNET(1,0.75)	ECVELNET(2,0.75)	ECVELNET(3,0.75)
		1.059(0.001)	1.067(0.002)	1.059(0.002)
CVLARS	TrLARS	ETrLARS(1)	ETrLARS(2)	ETrLARS(3)
0.963(0.002)	0.990(0.002)	1.076(0.002)	1.100(0.002)	1.083(0.002)
		ECVLARS(1)	ECVLARS(2)	ECVLARS(3)
		1.067(0.001)	1.046(0.003)	0.775(0.005)
CVALasso	TrAlasso	ETrAlasso(1)	ETrAlasso(2)	ETrAlasso(3)
0.899(0.002)	0.958(0.002)	1.004(0.003)	1.110(0.002)	1.100(0.002)
		ECVALasso(1)	ECVALasso(2)	ECVALasso(3)
		1.070(0.002)	1.086(0.002)	1.075(0.002)
CVSCAD	TrSCAD	ETrSCAD(1)	ETrSCAD(2)	ETrSCAD(3)
0.837(0.003)	0.891(0.003)	0.954(0.003)	0.969(0.003)	1.099(0.002)
		ECVSCAD(1)	ECVSCAD(2)	ECVSCAD(3)
		0.986(0.001)	1.014(0.002)	1.033(0.002)

**Table 2:** Efficiencies of trimmed mean methods with respect to the CVLasso for the model (7) with complexity index r/p = 0.15.

Method			
TrLasso	ETrLasso(1)	ETrLasso(2)	ETrLasso(3)
1.021(0.002)	1.015(0.003)	0.841(0.004)	0.759(0.004)
TrELNET(0.25)	ETrELNET(1,0.25)	ETrELNET(2,0.25)	ETrELNET(3,0.25)
1.023(0.003)	0.978(0.004)	0.835(0.004)	0.754(0.004)
ETrELNET(0.50)	ETrELNET(1,0.50)	ETrELNET(2,0.50)	ETrELNET(3,0.50)
1.026(0.002)	1.001(0.003)	0.841(0.004)	0.756(0.004)
TrELNET(0.75)	ETrELNET(1,0.75)	ETrELNET(2,0.75)	ETrELNET(3,0.75)
1.023(0.002)	1.009(0.003)	0.841(0.004)	0.756(0.004)
TrLARS	ETrLARS(1)	ETrLARS(2)	ETrLARS(3)
0.998(0.002)	1.049(0.003)	0.880(0.004)	0.733(0.004)
TrAlasso	ETrAlasso(1)	ETrAlasso(2)	ETrAlasso(3)
0.995(0.003)	0.971(0.003)	0.823(0.004)	0.763(0.004)
TrSCAD	ETrSCAD(1)	ETrSCAD(2)	ETrSCAD(3)
0.844(0.005)	1.017(0.003)	0.826(0.004)	0.771(0.004)

**Table 3:** Efficiencies of cross validated methods with respect to the CVLasso for the model (7) with complexity index r/p = 0.15.

Method			
CVLasso	ECVLasso(1)	ECVLasso(2)	ECVLasso(3)
-	0.974(0.003)	0.727(0.004)	0.671(0.004)
CVELNET(0.25)	ECVELNET(1,0.25)	ECVELNET(2,0.25)	ECVELNET(3,0.25)
1.033(0.002)	0.971(0.003)	0.722(0.004)	0.668(0.004)
CVELNET(0.50)	ECVELNET(1,0.50)	ECVELNET(2,0.50)	ECVELNET(3,0.50)
1.016(0.001)	0.977(0.003)	0.725(0.004)	0.670(0.004)
CVELNET(0.75)	ECVELNET(1,0.75)	ECVELNET(2,0.75)	ECVELNET(3,0.75)
1.006(0.000)	0.976(0.003)	0.726(0.004)	0.671(0.004)
CVLARS	ECVLARS(1)	ECVLARS(2)	ECVLARS(3)
0.953(0.003)	1.040(0.003)	0.822(0.004)	0.680(0.004)
CVALasso	ECVAlasso(1)	ECVAlasso(2)	ECVAlasso(3)
1.015(0.003)	1.073(0.004)	0.711(0.004)	0.732(0.004)
CVSCAD	ECVSCAD(1)	ECVSCAD(2)	ECVSCAD(3)
0.816(0.004)	0.875(0.004)	0.733(0.004)	0.682(0.004)

**Table 4:** Efficiencies of trimmed methods with respect to the CVLasso for the model (7) with r/p = 0.3.

Method			
TrLasso	ETrLasso(1)	ETrLasso(2)	ETrLasso(3)
1.056(0.002)	1.135(0.003)	1.092(0.005)	1.002(0.004)
TrELNET(0.25)	ETrELNET(1,0.25)	ETrELNET(2,0.25)	ETrELNET(3,0.25)
1.095(0.002)	1.130(0.003)	1.087(0.004)	0.997(0.004)
TrELNET(0.50)	ETrELNET(1,0.50)	ETrELNET(2,0.50)	ETrELNET(3,0.50)
1.073(0.002)	1.133(0.003)	1.092(0.005)	1.000(0.004)
TrELNET(0.75)	ETrELNET(1,0.75)	ETrELNET(2,0.75)	ETrELNET(3,0.75)
1.062(0.002)	1.133(0.003)	1.096(0.005)	1.003(0.004)
TrLARS	ETrLARS(1)	ETrLARS(2)	ETrLARS(3)
1.037(0.002)	1.146(0.003)	1.121(0.005)	0.957(0.004)
TrAlasso	ETrAlasso(1)	ETrAlasso(2)	ETrAlasso(3)
1.055(0.002)	1.104(0.003)	1.072(0.004)	1.006( 0.004)
TrSCAD	ETrSCAD(1)	ETrSCAD(2)	ETrSCAD(3)
0.836(0.004)	1.098(0.003)	1.054(0.004)	1.021(0.004)

**Table 5:** Efficiencies of cross validated methods with respect to the CVLasso for the model (7) with r/p = 0.3.

Method			
	ECVLasso(1)	ECVLasso(2)	ECVLasso(3)
-	1.050(0.002)	0.914(0.004)	0.873(0.004)
CVELNET(0.25)	ECVELNET(1,0.25)	ECVELNET(2,0.25)	ECVELNET(3,0.25)
1.060(0.002)	1.082(0.003)	0.920(0.004)	0.875(0.004)
CVELNET(0.50)	ECVELNET(1,0.50)	ECVELNET(2,0.50)	ECVELNET(3,0.50)
1.024(0.001)	1.063(0.002)	0.915(0.004)	0.874(0.004)
ECVELNET(0.75)	ECVELNET(1,0.75)	ECVELNET(2,0.75)	ECVELNET(3,0.75)
1.008(0.000)	1.055(0.002)	0.915(0.004)	0.873(0.004)
CVLARS	ECVLARS(1)	ECVLARS(2)	ECVLARS(3)
0.964(0.003)	1.106(0.002)	1.029(0.004)	0.883(0.004)
CVALasso	ECVAlasso(1)	ECVAlasso(2)	ECVAlasso(3)
1.004(0.003)	1.178(0.004)	0.913(0.004)	0.948(0.004)
CVSCAD	ECVSCAD(1)	ECVSCAD(2)	ECVSCAD(3)
0.888(0.003)	0.936(0.003)	0.899(0.004)	0.874(0.004)

## Data Availability

Not applicable.
